# A96 A QUALITY ASSESSMENT STUDY TO DETERMINE IF TISSUE ACQUISTION AND SPECIMEN HANDLING IMPACT THE DIAGNOSTIC YIELD OF ENDOSCOPIC ULTRASOUND-GUIDED FINE NEEDLE ASPIRATION OF SOLID MASS

**DOI:** 10.1093/jcag/gwad061.096

**Published:** 2024-02-14

**Authors:** S Khan, P Mathura, L Puttangunta, S Girgis, J Zhang, J Nilsson, S Wesilenko

**Affiliations:** Internal Medicine, University of Alberta, Edmonton, AB, Canada; Internal Medicine, University of Alberta, Edmonton, AB, Canada; Internal Medicine, University of Alberta, Edmonton, AB, Canada; Internal Medicine, University of Alberta, Edmonton, AB, Canada; Internal Medicine, University of Alberta, Edmonton, AB, Canada; Internal Medicine, University of Alberta, Edmonton, AB, Canada; Internal Medicine, University of Alberta, Edmonton, AB, Canada

## Abstract

**Background:**

Endoscopic ultrasound (EUS)-guided fine needle aspiration biopsy (FNAB) of solid mass lesions has a sensitivity and specificity between 50-100%. Tissue acquisition and specimen handling are factors that may contribute to this variability. At our institution, 3 endoscopists perform EUS. The aspirated material obtained is expressed onto slides for cytology (prepared by a nurse) and solid tissue fragments transferred into formalin. At the discretion of the endoscopist, aspirated material is collected in saline for cell block preparation.

**Aims:**

To assess the impact of tissue collection and specimen handling on diagnostic yield of EUS-FNAB of solid mass lesions.

**Methods:**

A chart audit was completed for all patients undergoing EUS-FNAB of solid mass lesions between January 1, 2022 and December 31, 2022. Descriptive statistics were completed. A definite diagnosis was considered when calculating the diagnostic yield.

**Results:**

A total of 184 patients (100 M, 84 F), mean age 64±13 years (range 14-89 years), underwent 200 EUS-FNABs by 3 endoscopists. Pancreatic masses were the most common indication in 118/200 (59%) cases. A 22-gauge FNAB needle was used in 189/200 (95%) cases. A total of 285 needle passes were performed in 149 cases (mean 1.9/case). In the remaining 51 cases (26%), the number of needle passes was not specified. Tissue samples were transported in formalin in 190 cases, on cytology slides in 170 cases, and in saline for cell block preparation in 41 cases. Overall, a definite diagnosis was achieved in 149/200 cases (75%). Stratifying for needle passes, a definite diagnosis was achieved in 22/39 (56%), 71/85 (84%), 20/24 (83%), and 1/1 (100%) cases that had 1, 2, 3, and 4 needle passes. Of the 51 cases with unspecified needle passes, a definite diagnosis was achieved in 35 cases (69%). The diagnostic yield obtained with saline for cell block was similar to that obtained with formalin and cytology slides (30/41 [73%] vs. 144/190 [76%] and 132/170 [78%]).

**Conclusions:**

Increasing the minimum number of needles passes for tissue acquisition to 3 per case may increase the diagnostic yield of EUS-FNAB. Documenting the number of needles passes in the endoscopy report is an important quality indicator. Cytology slides and tissue in formalin should be considered standard of care but aspirated material should continue to be used for cell block preparation. However, there is some concern that saline as a transport medium may de-vitalize the aspirated material, so it should be replaced with formalin to preserve tissue integrity.

**Funding Agencies:**

None

